# Interpretable instance disease prediction based on causal feature selection and effect analysis

**DOI:** 10.1186/s12911-022-01788-8

**Published:** 2022-02-26

**Authors:** YuWen Chen, Ju Zhang, XiaoLin Qin

**Affiliations:** 1 Chengdu Institute of Computer Applications, Chinese Academy of Sciences, Chengdu, China; 2 Chongqing Institute of Green and Intelligent Technology, Chinese Academy of Sciences, Chongqing, China; 3 University of Chinese Academy of Sciences, Beijing, China

**Keywords:** Causal effects, Interpretability, Feature selection, Disease prediction

## Abstract

**Background:**

In the big wave of artificial intelligence sweeping the world, machine learning has made great achievements in healthcare in the past few years, however, these methods are only based on correlation, not causation. The particularities of the healthcare determines that the research method must comply with the causality norm, otherwise the wrong intervention measures may bring the patients a lifetime of misfortune.

**Methods:**

We propose a two-stage prediction method (instance feature selection prediction and causal effect analysis) for instance disease prediction. Feature selection is based on the counterfactual and uses the reinforcement learning framework to design an interpretable qualitative instance feature selection prediction. The model is composed of three neural networks (counterfactual prediction network, fact prediction network and counterfactual feature selection network), and the actor-critical method is used to train the network. Then we take the counterfactual prediction network as a structured causal model and improve the neural network attribution algorithm based on gradient integration to quantitatively calculate the causal effect of selection features on the output results.

**Results:**

The results of our experiments on synthetic data, open source data and real medical data show that our proposed method can provide qualitative and quantitative causal explanations for the model while giving prediction results.

**Conclusions:**

The experimental results demonstrate that causality can further explore more essential relationships between variables and the prediction method based on causal feature selection and effect analysis can build a more reliable disease prediction model.

**Supplementary Information:**

The online version contains supplementary material available at 10.1186/s12911-022-01788-8.

## Background

Machine learning is becoming an increasingly important tool in healthcare. Some artificial intelligence systems have approached or even surpassed human experts in terms of cancer classification [1], cancer detection [2], diabetic retinopathy detection [3]. Artificial intelligence (AI) will, without doubt, help reshape the future of medicine.

**Fig. 1:**
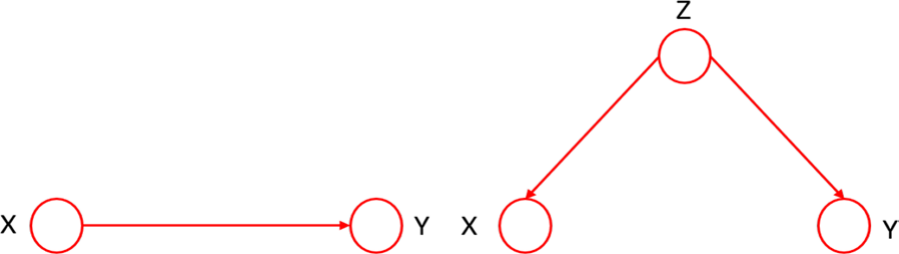
Causality diagram

However, the current methods that have been successfully applied to the above-mentioned medical problems are based only on association rather than causality. In statistics, people acknowledge that association does not logically imply causation [4, 5]. The relationship between correlation and causation was formalized by Reichenbach [6] as the common cause principle: if two random variables X and Y are statistically dependent, then one of the following causal explanations must be hold: (1) X is the direct cause of Y; (2) There is a random variable Z, which is the common reason for X and Y, as shown in Fig. 1. Therefore, compared with association, causality further explores more essential relationships between variables. The core task of causal inference is to reveal the causal relationship between different variables, which enables us to have the following abilities:(1) predict the outcome of a variable after intervention; (2) to estimate the impact of intervention and confounding factors; (3) Enable the model to predict unseen cases. If we think of medical treatment as an intervention and treat effect as an outcome, then these capabilities are needed in healthcare, but most existing approaches do not yet have them. Furthermore the particularities of the healthcare determines that the research method must comply with the causality norm, otherwise the wrong intervention measures may bring the patients a lifetime of misfortune. Therefore, causality plays a key role in developing truly intelligent medical algorithms.

In addition, with the rapid development of modern medical technology, more and more clinical observation data of patients are collected.However, this growth has a huge impact on the disease prediction model and the time consumption of patient detection and testing. In fact, contrary to popular belief, more variables is not synonymous of more useful information and a better prediction while in theory the more features are used the better. This can be easily explained by the fact that non relevant features induce over fitting and so decrease the performances and the generalization of the model. The traditional feature extraction can achieve good results in prediction and classification, but it describes the correlation between variables. Therefore, feature selection is one of the important steps to obtain a good prediction effect. In the case of cancer, for example, we need to know what causes it and what variables need to be used to cure it. In lung cancer, both smoking and coughing are contributing factors, but we need to know which the cause is and which the effect is. Because curing cough is not a cure for cancer as a result, banning smoking can prevent cancer because it is a direct cause.

**Fig. 2:**
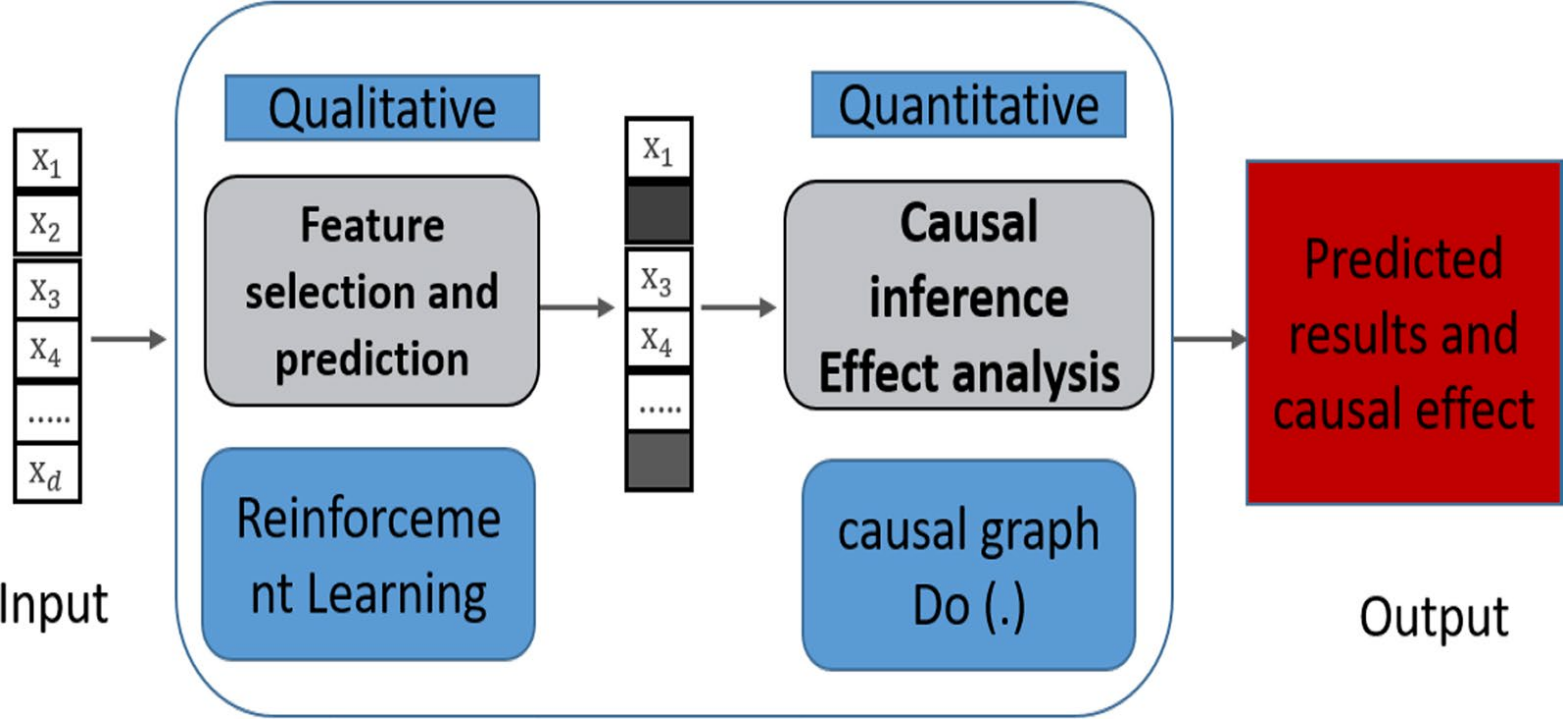
Two-stage model

Therefore, we propose a two-stage prediction method (instance feature selection prediction and causal effect analysis) for instance disease prediction, starting from knowledge in the medical field to infer the influence relationship between variables. So as to better understand the underlying mechanism behind the data set and evaluate the model more transparently. The model flow is shown in Fig. 2. Firstly, we use the reinforcement learning framework to design an interpretable qualitative instance feature selection prediction method based on the counterfactual. Then we take the counterfactual prediction network as a structured causal model and improve the neural network attribution algorithm based on gradient integration to quantitatively calculate the causal effect of selection features on the output results.

The main contributions of this paper can be summarized as follows: We use causal mediation analysis for causal feature selection for the first time, and design a framework for qualitative feature selection based on deep reinforcement learning. In addition, we improve the neural causal attribution algorithm based on the integration gradient, and perform quantitative causal average effect analysis on selected feature attributes in a more robust and interpretable way. Finally, we conducted experimental verification on public data, synthetic data and real medical data, which proved the effectiveness of the method.

### Related work

Machine learning has made great progress in the health [11–13].These apps must satisfy two conditions: (1) they must be causal and (2) they must be explainable. For example, in order to find the effect of a drug on a patient's health, it is necessary to estimate the causal relationship between the drug and the patient's health status. Moreover, in order for the results to be reliable to the doctor, it is necessary to explain how the decision was made.

Recently, interpretability models based on traditional methods have been studied in the following aspects. Attention network: neural network model based on attention mechanism can not only improve the accuracy of prediction, but also specifically show which input features or learning representation are more important for specific prediction, such as graph embedding [14] and machine translation [15, 16]. Representation learning: One goal of representation learning is to decompose features into independent latent variables that are highly correlated with meaningful patterns [11]. In traditional machine learning, methods such as PCA [17], ICA [18]and spectral analysis [19] are proposed to discover entangled components of data. Recently researchers have developed deep latent variable models such as VAE [20], InfoGan [10] and β-VAE [21] to learn to untangle the latent variables through variation reasoning. Locally interpretable model: LIME [9] is a representative and precursor framework that can estimate any black box prediction through a local proxy interpretable model. Saliency mapping: Originally developed by Simonyan et al. [22] as a "category saliency map for a particular image", it highlights the pixels of a given input image. These pixels are primarily concerned with identifying a particular category of label for an image. To extract these pixels, a back propagation algorithm can traverse (deconvolution) to find the derivative of the weight vector, and the magnitude of the derivative indicates the importance of each pixel to the category score. Other researchers have used similar concepts to deconvolve predictions and show the location of input images that strongly influence neuronal activation [23–25]. Although these methods are popular tools for interpretability, Adebayo et al. [26] and Ghorbani et al. [27] argue that relying on visual assessments is insufficient and may be misleading.

In addition, feature selection based on information theory also has corresponding work. Fast correlation-based filter (FCBF) was proposed by Lei Yu and Huan Liu in [33]. This paper mainly proposes to use symmetric uncertainty instead of information gain to measure whether a feature is related to classification C or redundant. Minimum redundancy and maximum relevance (MRMR) algorithm [34] is a feature selection algorithm for single label data. The main purpose of this typical feature attribute selection algorithm is to select m features from n features and ensure that the feature subset can keep the classification results of data samples close to or even better than those of all features. Brown et al. [35] present a unifying framework for information theoretic feature selection, bringing almost two decades of research on heuristic filter criteria under a single theoretical interpretation. This paper mainly focuses on the feature selection of causality. Counterfactual analysis and causal inference have gained a lot of attention from the interpretable machine learning field. Research in this area has mainly focused on generating counterfactual explanations from both the data perspective [28, 29] as well as the components of a model [30, 31].Pearl [32] introduces different levels of said interpretability and argues that generating counterfactual explanations is the way to achieve the highest level of interpretability. Therefore, this paper attempts to select causal features based on neural network and causal reasoning. The relevant methods are described as follows.

## Methods

The study protocol was approved by the Institutional Ethics Committee of Southwest Hospital of Third Military Medical University (No. KY201936.). We confirm that all methods were performed in accordance with the relevant guidelines and regulations.

In order to provide a common understanding throughout the text, this section describes the concept of Structural Causal Model, Do-operator, and Integral gradient.

### Structural causal model (SCM)

The structural causal model (SCM) [4] is a 4-tuple $\left(\mathrm{X},\mathrm{U},\mathrm{f},{P}_{u}\right),$ in which X is a set of finite endogenous variables, usually observable random variables in the system. U is a finite set of exogenous variables, which are generally regarded as unobserved variables or noise variables. F is a set of functions $[{f}_{1},{f}_{2,}\dots {f}_{n}]$, where n refers to the cardinality of the set X. These functions define the causal mechanism, such as $\forall {x}_{i}\in X,{x}_{i}={f}_{i}(par,{U}_{i})$. Par $\in \mathrm{X}-\left\{{x}_{i}\right\}$ and ${U}_{i}\in U$, ${P}_{u}$ defines the probability distribution on U. Structural causal models represent causal dependencies using graphical models that provide an intuitive visualization by representing variables as nodes and relationships between variables as edges in a graph. Graphical models serve as a language for structuring and visualizing knowledge about the world and can incorporate both data-driven and human inputs. Counterfactuals enable the articulation of something there is a desire to know, and structural equations serve to tie the two together.

### The do-operator and interventional

**Fig. 3:**
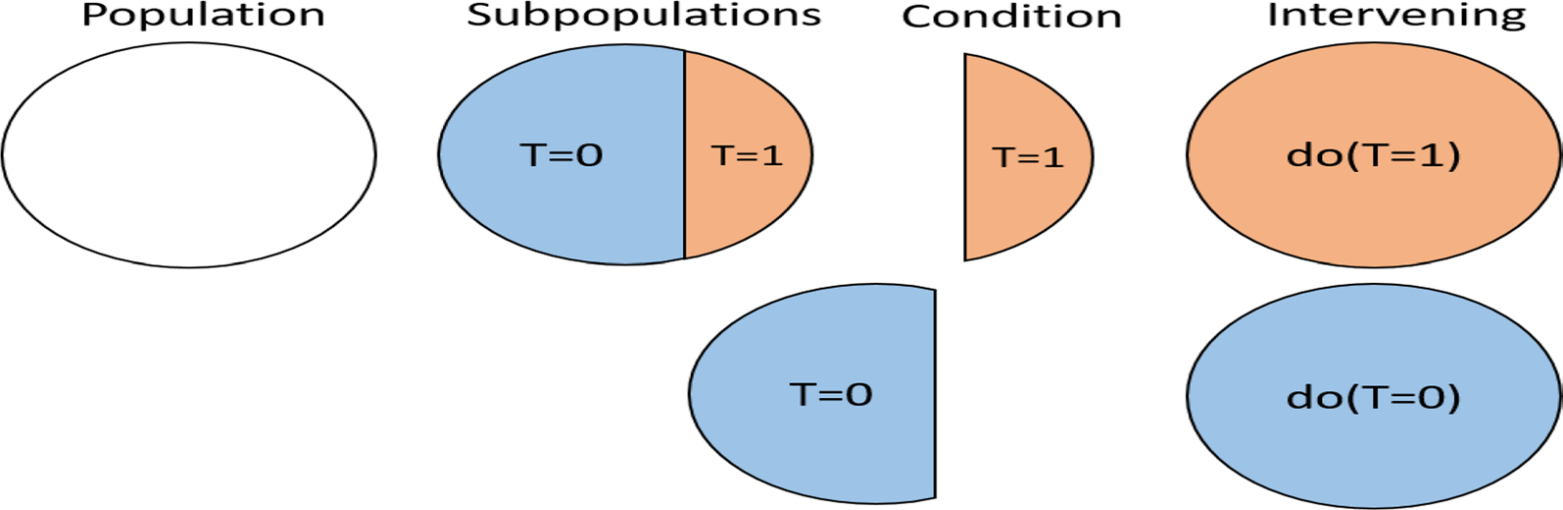
Difference between conditional distribution and intervention distribution

Conditional probability is different from do-operator and intervention distribution. The condition of T = t only means that we focus our attention on the people receiving treatment t. In contrast, intervention involves treating the entire population. This is illustrated in Fig. 3. We use the do-operator to express intervention: do (T = t), which is a commonly used notation in graph causal models and is equivalent to the latent result notation [7]. When the treatment is binary, the average treatment causal effect is as in formula (1):

1 $${\text{E}}\left[ {{\text{Y}}|{\text{do}}\left( {{\text{T}} = {1}} \right)} \right] - {\text{E}}\left[ {{\text{Y}}|{\text{do}}\left( {{\text{T}} = 0} \right)} \right]$$

### Integral gradient

Suppose the function $\mathrm{F}:{R}^{n}\to [\mathrm{0,1}]$ represents a neural network. $\mathrm{x}\in {R}^{n}$ is the neural network input vector, and ${x}^{\mathrm{^{\prime}}}\in {R}^{n}$ is the baseline input. Consider the linear path from the baseline ${x}^{\mathrm{^{\prime}}}$ to the input x in the space ${R}^{n}$, calculate the gradients of all points along the path, and obtain the integral gradient by accumulating these gradients. Specifically, the integral gradient is defined as the integral path of the gradient along a straight line path from the baseline ${x}^{\mathrm{^{\prime}}}$ to the input x. The integral gradient of input x and baseline ${x}^{\mathrm{^{\prime}}}$ along the ith dimension is defined as follows, where $\frac{\partial F(x)}{{x}_{i}}$ is the gradient of F(X) along the ith dimension.

2 $${IntegratedGrad}_{i}\left(x\right)=({x}_{i}-{x}_{i}^{\mathrm{^{\prime}}})\times {\int }_{\alpha =0}^{1}\frac{\partial F({x}^{\mathrm{^{\prime}}}+\alpha \times (x-{x}^{\mathrm{^{\prime}}}))}{\partial {x}_{i}}d\alpha$$

### Problem formulation

This work attempts to solve the following problems: "How to achieve qualitative selection of causal features and quantitative causal effect analysis through deep neural networks. That is, how to flexibly select different numbers of causal feature variables for each sample and quantify the causal effects of the selected causal variables on specific output neurons." Therefore, we propose a two-stage causal feature selection prediction and effect analysis method. This is shown in Fig. 2. The details are as follows:

Let $\upchi ={\upchi }_{1 }\times {\upchi }_{2}\times \dots \dots {\upchi }_{d}$ is the d-dimensional feature space, and $\Upsilon =\{1,\dots .\mathrm{c}\}$ is the discrete label space. Let $\mathrm{X}$ represent the collection of all observation attributes of the patient,$\mathrm{D}={\{({X}_{i},{Y}_{i})\}}_{i=1}^{n}$ represents a collection of patient clinical data,${X}_{i}\in\upchi$ Clinical observation data of patient i,${Y}_{i}\in \mathrm{\Upsilon label}$ of patient i. Let Z be a subset of X, representing some of the selected dimensional features. Among them, we use the ${Z}_{opt}$ to represent the optimal predictive feature set, and ${Z}_{\sim opt}$ to represent the non-optimal feature set. Then our problem is to find the optimal ${Z}_{opt}$ when predicting the label of each patient, and then analyze the causal effect of the ${Z}_{opt}$.

### Qualitative causal feature selection

**Fig. 4:**
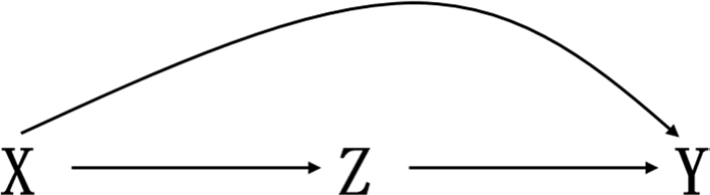
Causality diagram of patient data. X: observation data set, Z: feature subset, Y target label

According to medical knowledge, we can draw the following causality diagram. It can be seen from the Fig. 4 that Z can be regarded as an mediation variable of X and Y, which is unobservable and is a hidden variable required by the model.

If Z is the optimal predictor subset mediator variable, that is, Z is required to be completely mediator and the influence of X on Y is completely determinable by Z. In other words, it is required to maximize the natural indirect effect (NIE) of formula (3).

3 $$\mathrm{NIE}=\mathrm{P}({Y}_{Z={z}_{opt}}=1|\mathrm{do}(\mathrm{X}=\mathrm{All}))-\mathrm{P}({Y}_{{Z=Z}_{\sim opt}}=1|\mathrm{do}(\mathrm{X}=\mathrm{All}))$$

where do(X = All) means that X takes all the observation attributes set.

The output space size of the feature optimal subset Z increases exponentially with the size of the feature space. In order to facilitate optimization, we fix ${Z}_{\sim opt}$ as the full feature subset ${Z}_{\sim opt}=X$ and only intervene Z = ${Z}_{opt}$, Let Z be a completely mediator, and then minimize formula (4), which is consistent with the definition of relevant feature selection.

4 $${NIE}^{\mathrm{^{\prime}}}=\mathrm{P}({Y}_{Z={z}_{opt}}=1|\mathrm{X})-\mathrm{P}({Y}_{Z=X}=1|\mathrm{X})$$

**Fig. 5:**
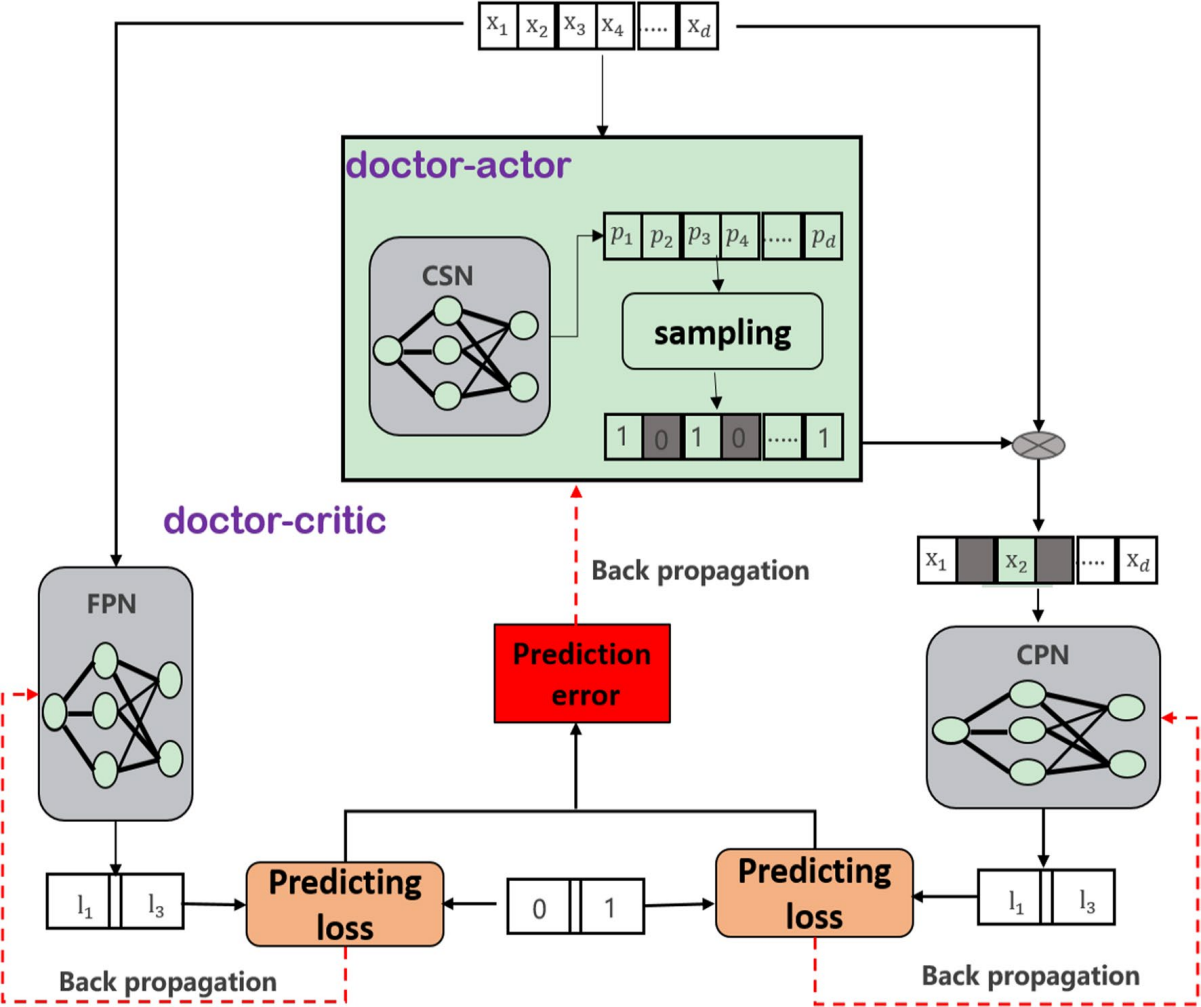
The instance is input to the selector network, which outputs the selection probability vector. The selection vector is then sampled based on these probabilities. Then, the prediction network receives the selected features and makes predictions, and the baseline network gives the entire feature vector and makes predictions. Each of these networks is back-propagated training using real labels. Then subtract the loss of the baseline network from the loss of the prediction network, which is used to update the selector network. *CPN* counterfactual prediction network, *CSN* counterfactual selection network, *FPN* fact prediction network

There is a natural correspondence between interventions in causal reasoning and actions taken in reinforcement learning. Therefore, we define the first half of formula (4) as an actor that performs counterfactual selection prediction on the ${Z}_{opt}$. The latter part is defined as a critical, which predicts facts and evaluates actors. We use the Kullback–Leibler (KL) divergence[] to convert constraint (4) into a soft constraint to maximize the causal effect of mediation Z in formula (5).The model is shown in Fig. 5.

5 $$\mathrm{L}\left(\mathrm{S}\right)={E}_{z\sim Pz}[KL(({Y}_{Z={z}_{opt}}|\mathrm{X})|({Y}_{Z=x}|\mathrm{X}))]$$

Therefore, we use the three neural network to fit the causal structure equation function to optimize the formula (4).${f}^{\theta }$: counterfactual prediction network (${Z}_{opt}\to Y$), ${f}^{\gamma }$:fact prediction network ($\mathrm{X}\to Y$), ${f}^{\vartheta }$: counterfactual selection network ($\mathrm{X}\to {Z}_{opt}$).

### Counterfactual prediction network

We design ${f}^{\theta }$ as a counterfactual predictor network, accepting the selected feature vector of the counterfactual as input, and output the probability distribution on the c-dimensional output space. The loss function of the network is as follows:

6 $${l}_{1}(\theta )=- {E}_{\left(x,y\right)\sim {p}_{xy},z\sim {\pi }_{\vartheta (x,.)}}\left[\sum_{i=1}^{c}{y}_{i}\mathrm{log}({f}_{i}^{\theta }({x}^{\left(z\right)},z))\right]$$

where ${y}_{i}$ is the ith component code of y, and ${\pi }_{\vartheta }$ is the distribution of the counterfactual selection network, which is defined in the next section. ${f}^{\theta }$ is implemented by a fully connected neural network.

### Factual prediction network

We design ${f}^{\gamma }$ as the fact prediction network, which is called critical. ${f}^{\gamma }$ is designed as a fully connected neural network. The network uses all observed patient data to make direct predictions. The loss function of the network is as follows:

7 $${l}_{2}(\gamma )=- {E}_{\left(x,y\right)\sim {p}_{xy},}\left[\sum_{i=1}^{c}{y}_{i}\mathrm{log}({f}_{i}^{\gamma }(x))\right]$$

Whether it is a factual prediction network or a counterfactual prediction network, our goal is to make the prediction consistent with the ground truth, and to maximize the probability of choosing the real optimal subset Z. Therefore, we fix $\uptheta ,\upgamma$, and define the total loss function of the two networks as:

8 $$\widehat{l}(x,z)=-\left[\sum_{i=1}^{c}{y}_{i}\mathrm{log}\left({f}_{i}^{\theta }\left({x}^{\left(z\right)},z\right)\right)-\sum_{i=1}^{c}{y}_{i}\mathrm{log}({f}_{i}^{\gamma }(x))\right]$$

### Counterfactual selection network

We design ${f}^{\vartheta }$ as the fact counterfactual selection network. ${f}^{\vartheta }$:$\mathrm{X}\to {\{\mathrm{0,1}\}}^{d}$, The network outputs the selection probability of each feature. The probability of a given feature selection vector $\mathrm{s}\in {\{\mathrm{0,1}\}}^{d}$ is:

9 $${\pi }_{\vartheta }\left(x,z\right)={\Pi }_{i=1}^{d}{f}_{i}^{\vartheta }{(x)}^{{s}_{i}}{({1-f}_{i}^{\vartheta }(x))}^{1-{s}_{i}}$$

Define the loss function of the counterfactual selection network:

10 $${l}_{3}={E}_{\left(x,y\right)\sim {p}_{xy}}\left[\sum_{s\in {(\mathrm{0,1})}^{d}}{\pi }_{\vartheta }\left(x,z\right)(\widehat{l}\left(x,z\right)+\lambda {\Vert { f}^{\vartheta }\Vert }_{0})\right]$$

We can use the BP back propagation algorithm to train the three neural networks end-to-end, by combining the above three loss functions as shown in Fig. 5. We input patient observation data into the trained model, and then we can get the optimal subset of the feature and the prediction result.

### Analysis of quantitative causal effects of selected features

**Fig. 6:**
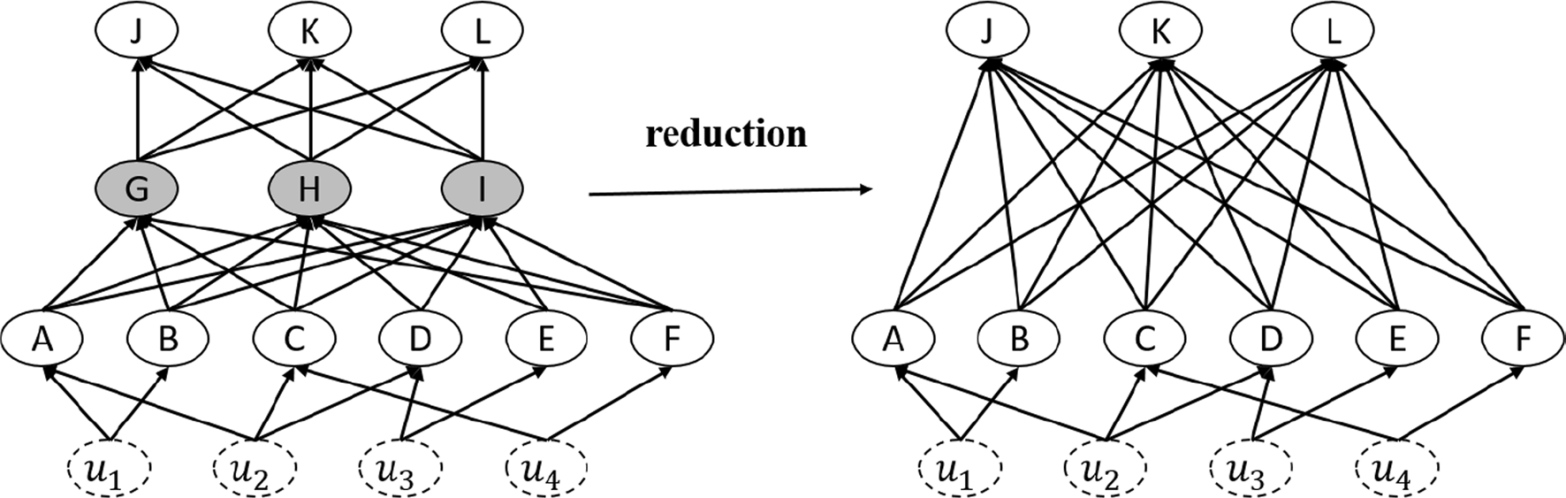
Feed-forward neural network as SCM. The dotted circles represent exogenous random variables that can be used as common causes for different input feature

Chattopadhyay [8] simplified the multilayer neural network into a two-layer causal structure model, and calculated the average causal effect(ACE) of input neurons on output neurons. Figure 6. Based on this work, this section uses integral gradient to improve the calculation of the average causality effect of qualitative feature selection.

Given a neural network with input ${l}_{1}$ and output ${l}_{n}$, we hence measure the ACE of an input feature ${x}_{i}=\alpha \in {l}_{1}$ with value α on an output feature $\mathrm{y}\in {l}_{n}$ as: (See the Additional file 1: Appendix for specific definitions)

11 $${ACE}_{do({x}_{i}=\alpha )}^{y}=\mathrm{E}[\mathrm{y}|\mathrm{do}({x}_{i}=\alpha )]-{baseline}_{{x}_{i}}$$

We define the baseline value of each input neuron as:

12 $${baseline}_{{x}_{i}}={E}_{{x}_{i}}[{E}_{y}[y|do({x}_{i}=\alpha )]]$$

In the implementation, we evaluate the baseline by evenly perturbing the input neuron ${x}_{i}$ from a fixed interval of [${low}_{i},{high}_{i}$] and calculating the intervention expected value.

Consider an output neuron y in the reduced SCM ${M}^{\mathrm{^{\prime}}}(\left[{l}_{1},{l}_{n}\right],U,{f}^{\mathrm{^{\prime}}},{P}_{u})$ obtained by marginalizing out the hidden neurons in a given neural network ${M}^{\mathrm{^{\prime}}}(\left[{l}_{1},{l}_{n}\right],U,{f}^{\mathrm{^{\prime}}},{P}_{u})$. The causal mechanism can be written as $\mathrm{y}={f}_{y}^{\mathrm{^{\prime}}}$(${x}_{1},{x}_{2}\dots .{x}_{k}$), where ${x}_{i}$ refers to neuron i in the input layer, and k is the number of input neurons. If we perform a $do({x}_{i}=\alpha )$ operation on the network, the causal mechanism is given by $\mathrm{y}={f}_{y|do({x}_{i=\alpha })}^{\mathrm{^{\prime}}}$(${x}_{1},{x}_{2}\dots .{x}_{k}$). Let ${\mu }_{j}=E[{x}_{j}|do({x}_{i}=\alpha )]\forall {x}_{j}\in {l}_{1}$. Now, the second-order Taylor’s expansion of the causal mechanism ${f}_{y|do({x}_{i=\alpha })}^{\mathrm{^{\prime}}}$ around the vector $\upmu =[{\mu }_{1},{\mu }_{2}\dots .{\mu }_{k}]$ is given by (recall ${l}_{1}$ is the vector of input neurons):

13 $${f}_{y}^{\mathrm{^{\prime}}}({l}_{1})\approx {f}_{y}^{\mathrm{^{\prime}}}\left(\mu \right)+{\nabla }^{T}{f}_{y}^{\mathrm{^{\prime}}}\left(\mu \right)({l}_{1}-\mu )+ \frac{1}{2}{({l}_{1}-\mu )}^{T}{\nabla }^{2}{f}_{y}^{\mathrm{^{\prime}}}\left(\mu \right)({l}_{1}-\mu )$$

Take expectations on both sides at the same time (marginalize other input neurons):

14 $${E[f}_{y|do({x}_{i=\alpha })}^{\mathrm{^{\prime}}}\left(\left({l}_{1}\right)\right]\approx {f}_{y}^{\mathrm{^{\prime}}}\left(\mu \right)+\frac{1}{2}{Tr{\nabla }^{2}{f}_{y}^{\mathrm{^{\prime}}}\left(\mu \right)E[({l}_{1}-\mu )}^{T}\left({l}_{1}-\mu \right)\left|do\left({x}_{i}=\alpha \right)\right]$$

We now only need to calculate the individual interventional means µ and the interventional covariance between input features ${E[({l}_{1}-\mu )}^{T}({l}_{1}-\mu )|do({x}_{i}=\alpha )]$ to compute formula (14). We assume that the input neuron after intervention is d-separated from all other input neurons (See Additional file 1: Appendix for details).Therefore, the intervention mean and covariance are equal to the observed mean and covariance, respectively.

The formula (14) needs to calculate the second-order Hessian matrix of ${f}_{y|do({x}_{i=\alpha })}^{\mathrm{^{\prime}}}$. There is gradient saturation in the deep neural network training, and the average causal effect calculated according to formula (14) may also be saturated, that is, we don't get effective average causal effect. Therefore, we introduce the integral gradient to replace the solution of the gradient in formula 14. The average result of the gradient of each point on the straight line from ${x}_{i}$ to ${\widehat{x}}_{i}$. Because we're taking into account the gradients of all the points along the path, we're no longer constrained by the fact that the gradient at one point is zero. In the implementation we chose the zero vector as the benchmark. The first-order integral gradient calculation formula is as follows:

15 $${\nabla f}_{y}^{\mathrm{^{\prime}}}\left(\mu \right)=\left|\left[\frac{1}{n}\sum_{k=1}^{n}\left({\nabla }_{\gamma }{f}_{y}^{\mathrm{^{\prime}}}\left(\gamma \left(a\right)\right){|}_{\gamma \left(a\right)=\left(1-a\right)x+a\widehat{x},a=\frac{k}{n}}\right)\right]{[\widehat{x}-x]}_{i}\right|$$

Based on the results of the first-order integral gradient, we can directly calculate the second-order Hessian matrix of Formula (14) and calculate the average causal effect of input neurons on output neurons.

Therefore, combining the above two-stage model, we can perform feature selection prediction and average causal effect analysis for each patient. See the detailed experimental results in the following section.

## Results and experiments

In this section, we experimentally evaluate the proposed model on synthetic data, open source data, and real world medical data. We evaluate our performance both at the relevance of feature selection and the accuracy of prediction. We compare our qualitative feature selection model with two methods: LIME [9], and Shapley [10].compare our prediction model with XGBOOST and LASSO regularized linear model. In order to verify the effectiveness of the model, we also compare the open source data and real medical data with neural and support vector machine (SVM).Finally, we conduct quantitative analysis on the causal effect of the selected features.

The experimental environment of this article was based on the server: Ubuntu 16.04 LTS was used as the operating system with Intel Xeon e5-2650 V4 processor and Nvidia GTX 1080 Ti GPU, the memory is 63 GB. Pytorch was used to build the model, and Python3.6 was used as the programming tool.

### Synthetic data experiments

We firstly verify the effectiveness of model feature selection based on synthetic data. The input features are generated from an 11-dimensional Gaussian distribution with no correlations across the features. The label Y is sampled as a Bernoulli random variable with $\mathrm{P}\left(\mathrm{Y}=0|\mathrm{X}\right)=\frac{logit(X)}{1+logit(X)}$ where logit(X) is varied to create 3 different synthetic datasets:

16 $$\mathrm{Datasets}1:\mathrm{exp}({X}_{0}{X}_{1})$$

17 $$\mathrm{Datasets}2:\mathrm{exp}(\sum_{i=2}^{5}{X}_{i}^{2}-4)$$

18 $$\mathrm{Datasets}3:-10\times \mathrm{sin}2{X}_{6}+2\left|{X}_{7}\right|+{X}_{8}+\mathrm{exp}(-{X}_{9})$$

For each of Datasets-1 to Datasets-3 We generate 40,000 samples, 20,000 samples for training and 20,000 samples for testing. When focusing on feature selection, the performance indicators we use are true positive rate (TPR) (the higher the better) and false discovery rate (FDR) (the lower the better) to measure the performance of the method. We use the area under the receiver operating characteristic curve (AUROC), the area under the accuracy recall curve (AUPRC) and accuracy when the focus is prediction.

In this experiment we analyze the effect of using feature selection as a pre-processing step for prediction. We first perform feature selection and then train a 3-layer fully connected network to perform predictions on top of the (feature-selected) data. In this setting we compare the two feature selection methods (Lime and shapely) Furthermore, we also compare with the predictive model with XGBOOST and LASSO regularized linear model.

As demonstrated by Table 1, both TPR and FDR of our model are substantially superior to the Lime and Shapely methods. TPR and FDR of dataset 1 are 100% and 0. TPR and FDR of dataset 2 are 100% and 0. TPR and FDR of dataset 3 are 92% and 0. It indicates that our method is capable of detecting relevant features. In order to verify the effectiveness of the selection features of the counterfactual prediction network, we conducted experiments based on the counterfactual prediction network (Model proposed in this paper), the Factual prediction network, XGBOOST and LASSO respectively. The experimental results are shown in the Table 2.As can be seen in Table 2, there is a significant performance improvement when discarding all of the irrelevant features. However, neither of the feature selection methods (XGBOOST and LASSO) are capable of achieving this improvement.

**Table 1:** Feature selection result for synthetic datasets

Dataset	Dataset1		Dataset2		Dataset3	
Metrics (%)	TPR	FDR	TPR	FDR	TPR	FDR
Our model	100	0	100	0	92	0
LIME	13.8	86.2	100	0	98.1	1.9
Shapley	60.4	39.6	93.3	6.7	65.2	9.1

**Table 2:** Prediction performance results

Dataset	XGBOOST	With LASSO	Factual prediction network	Counterfactual prediction network
AUROC				
Dataset1	.574 ± 0.10	.498 ± 0.06	.681 ± 0.02	.693 ± 0.06
Dataset2	.872 ± 0.03	.823 ± 0.61	.864 ± 0.61	.877 ± 0.03
Dataset3	.899 ± 0.01	.862 ± 0.03	.890 ± 0.03	.911 ± 0.02
AUPRC				
Dataset1	.577 ± 1.02	.499 ± 0.08	.681 ± 0.04	.694 ± 0.03
Dataset2	.878 ± 0.31	.591 ± 0.37	.861 ± 0.21	.886 ± 0.04
Dataset3	.904 ± 0.04	.890 ± 0.02	.890 ± 0.05	.905 ± 0.02

**Fig. 7:**
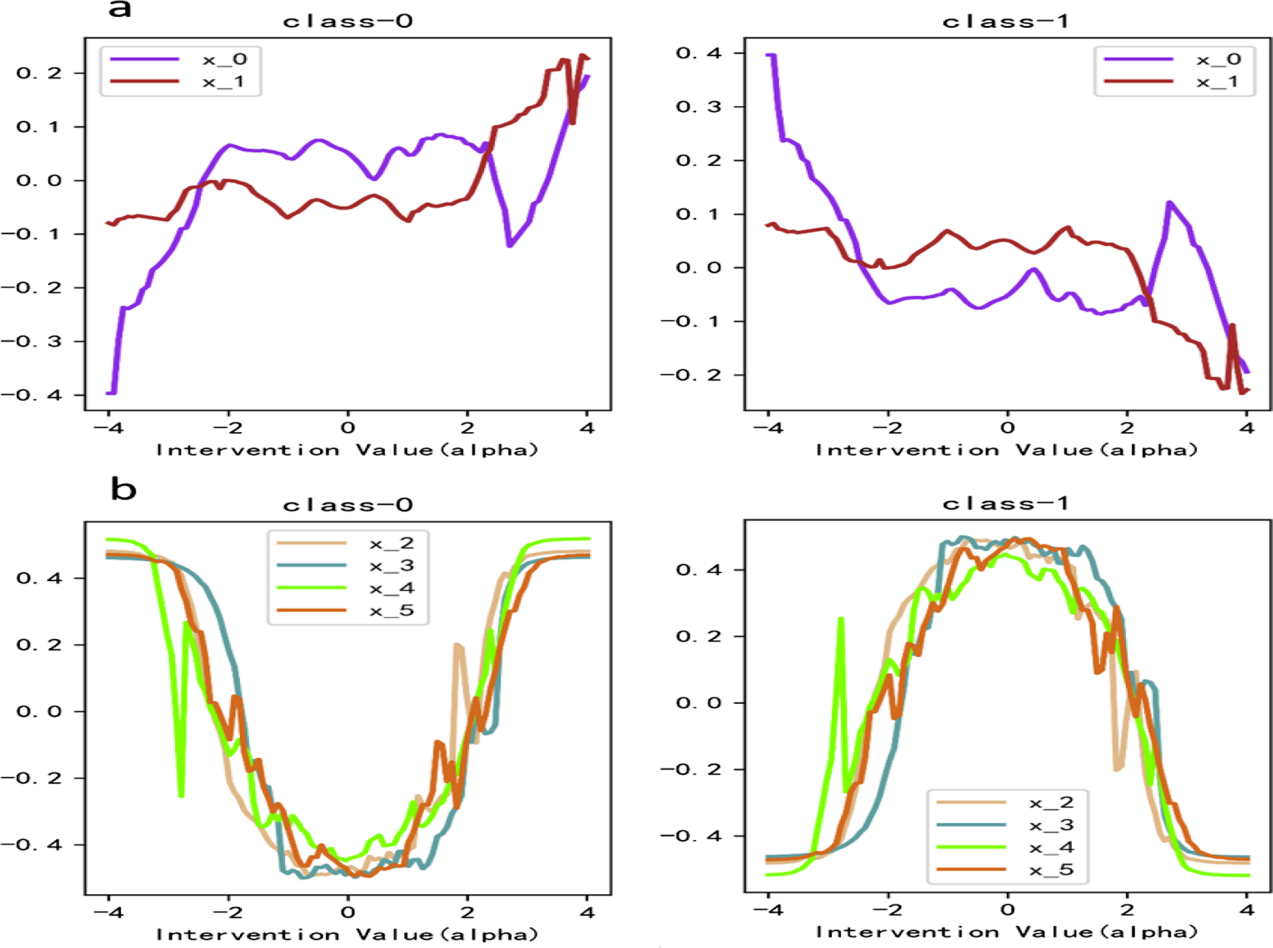
Causal effect analysis diagram. **a** Datasets1 and **b** Datasets2

Figure 7 describes the causal effect analysis diagram of the dataset sample. As can be seen in Fig. 7a, the selection of X0 and X1 in our model indicates the correctness of the selection of causal features. X0 and × 1 are positively correlated with the average causal effect of negative classification results, and vice versa. The attribution curve exactly fits the data generation process. Figure 7b also shows the attribution process. From the data generation formula (17), we can see that when X < 0, the probability of a sample being classified as negative is monotonically decreasing, and when x > 0, the probability of being classified as negative is monotonically increasing. The figure clearly describes that the model chooses × 2, × 3, × 4, and × 5 as prediction features. Interfering with these four feature values, the corresponding causal effects are consistent with the monotonicity of the data generation process, indicating the effectiveness of the model designed in this paper for the quantitative analysis of causal effects. It can also be seen that the model captures the causal relationship between each variable and Y well. Although the model chooses the variable × 9, it can be seen that the average causal effect of × 9 on y is basically 0. It shows that the variable × 9 has no causality with the prediction task.

### Obesity levels based on eating habits and physical condition data set

In this section we use open source healthcare data to perform a series of further experiments. This dataset include data for the estimation of obesity levels in individuals from the countries of Mexico, Peru and Colombia, based on their eating habits and physical condition. The data contains 17 attributes and 2111 records. 77% of the data was generated synthetically using the Weka tool and the SMOTE filter, 23% of the data was collected directly from users through a web platform. All data was labeled and the class variable was created with the values of: normal and abnormal in this experiment (See the Additional file 1: Appendix for the specific attributes of the data set).

**Fig. 8:**
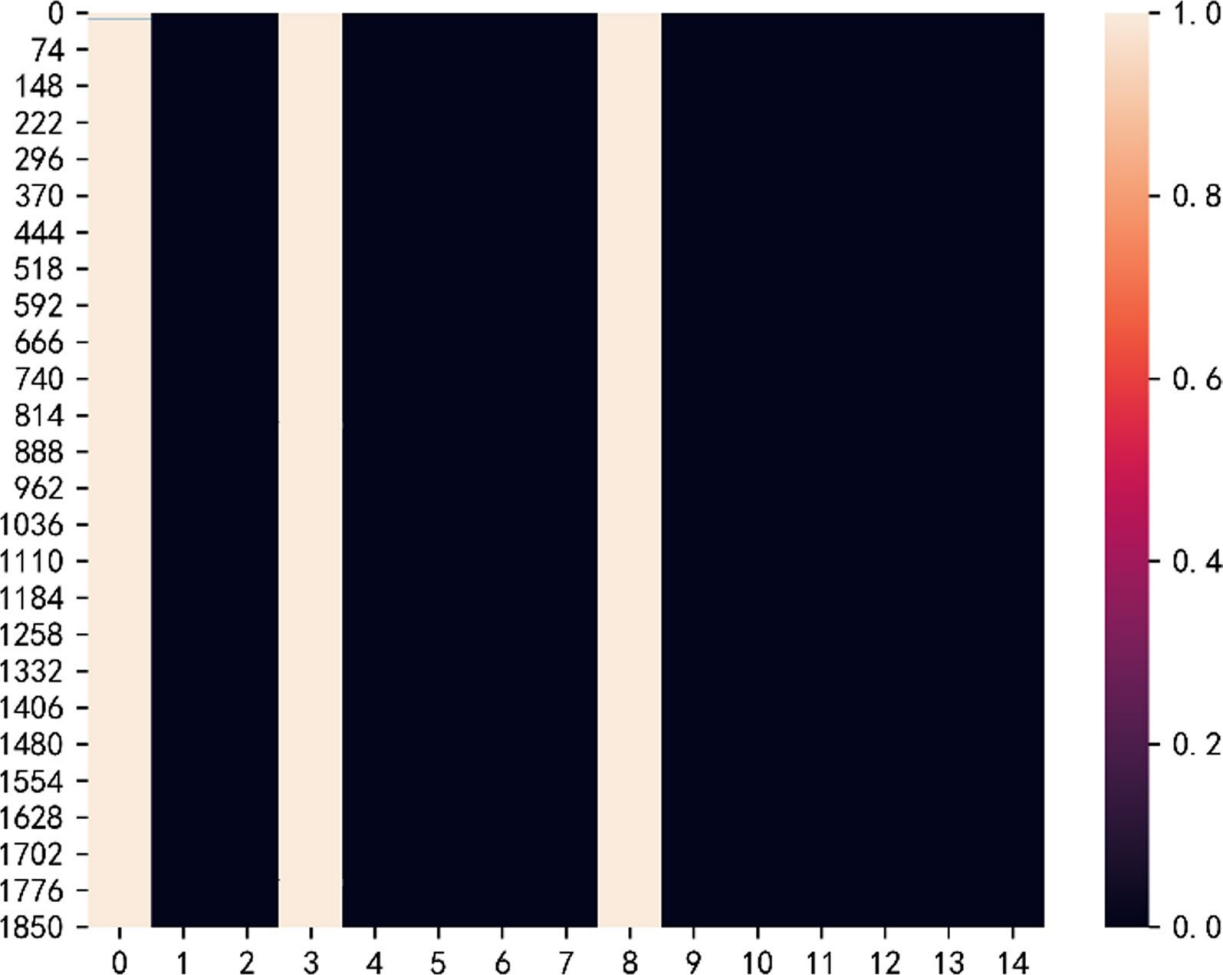
Feature selection probabilistic heat map

It can be seen from Table 3 that our proposed model is basically consistent with the performance of the full feature prediction method in terms of health prediction ability. The reason for our analysis may be that the number of features is inherently small and there is a strong correlation between the selected features and the predicted labels, so the advantages of our feature selection model have not been reflected. In addition, in the experiment, we drew a heat map of the feature selection probability of test patients. Figure 8 shows that the main reason for the model to predict patients is weight, FHWO, CAEC and FAF variables.

**Table 3:** Prediction performance results

Datasets	Method	AUROC	AUPRC	ACC
Obesity	XGBOOST	0.898 ± 0.04	0.915 ± 0.02	0.855 ± 0.06
	LR With LASSO	0.840 ± 0.05	0.92 ± 0.03	0.834 ± 0.01
	Neural network	0.839 ± 0.02	0.89 ± 0.01	0.831 ± 0.01
	SVM	0.810 ± 0.01	0.83 ± 0.02	0.82 ± 0.02
	With our model	0.840 ± 0.04	0.900 ± 0.02	0.836 ± 0.06

**Fig. 9:**
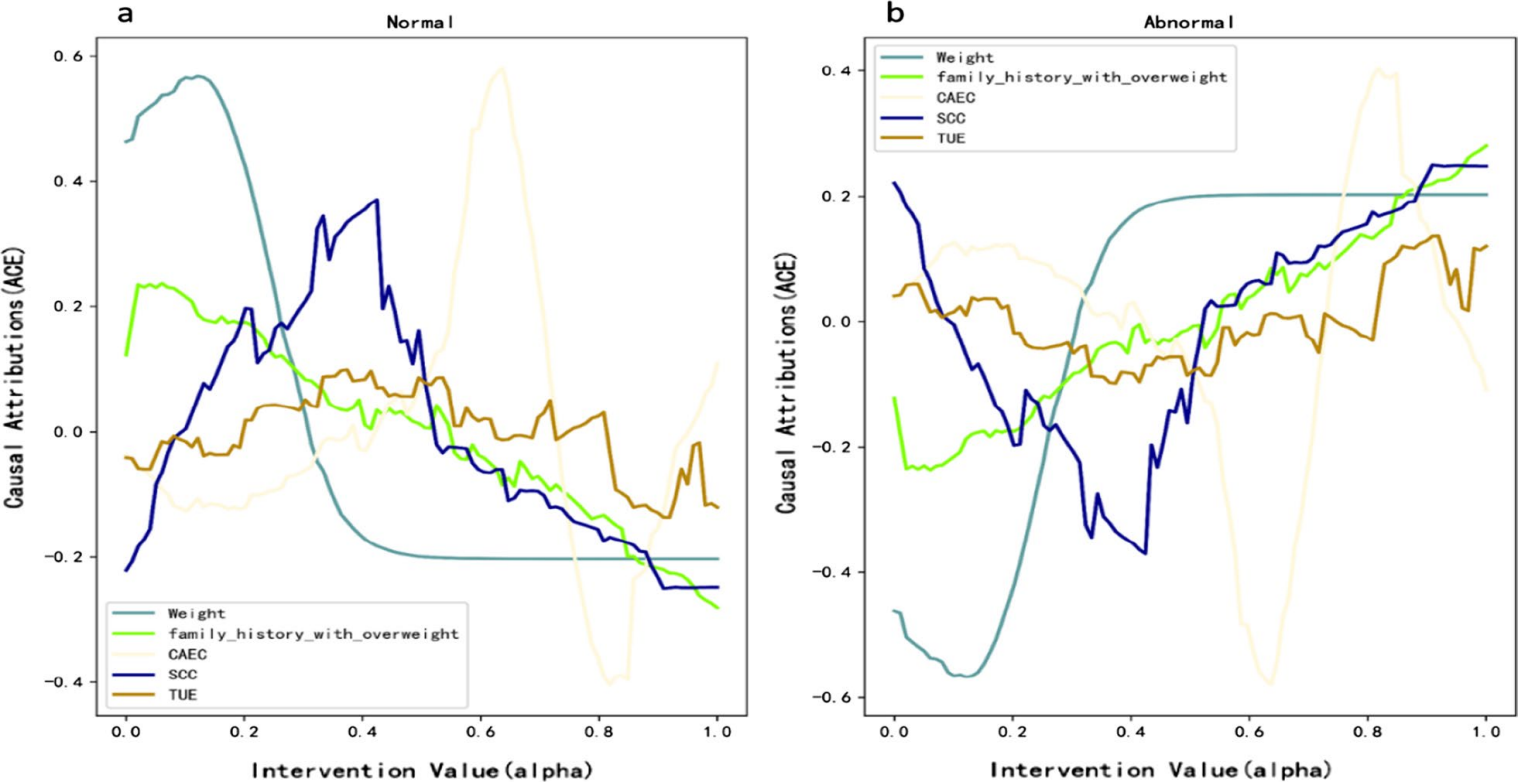
Causal effect analysis diagram. **a** Normal and **b** abnormal

Figure 9a, b depict average causal effect for the two classes and selected features. These plots easily reveal that smaller weight is positively causal (ACE ≥ 0) for Normal class and negatively causal (ACE < 0) for Abnormal class. Consumption of food between meals (CAEC) is a discrete value (No:0, Sometimes:1, Frequently:2, Always:3). It can be easily seen from the figure that frequently Consumption of food between meals is negatively causal for normal class and positively causal for Abnormal class. Therefore, from the results of causal effect analysis, the conclusions of the model are consistent with common medical knowledge.

### Heart failure data

**Fig. 10:**
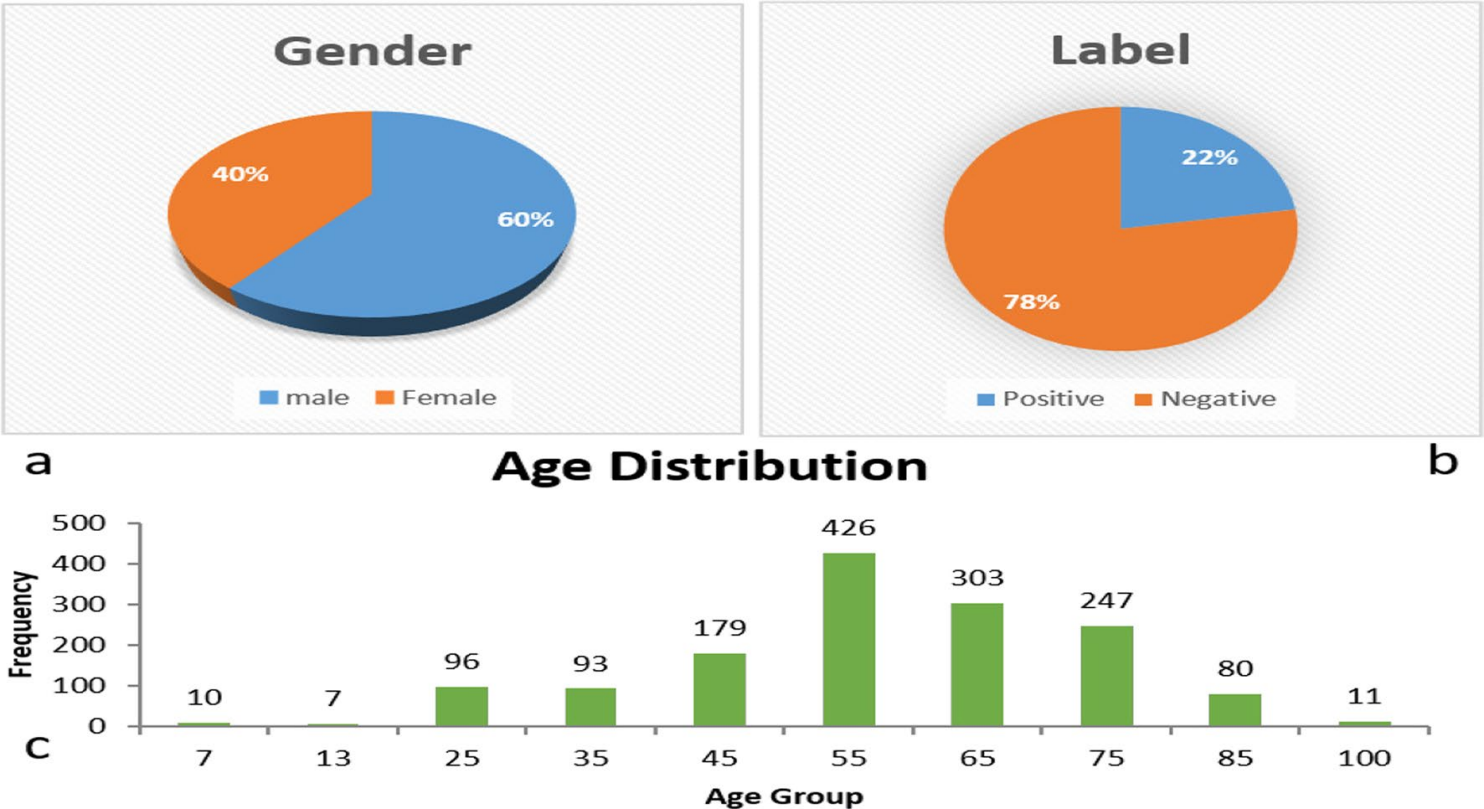
Heart failure data set distribution. **a** Gender, **b** label, **c** age

In this section, we use heart failure datasets to perform a series of further experiments. The data has 1452 patients each with 84 measured features, which were collected from surgery patient in hospital (the First Affiliated Hospital of Military Medical University of the Army) of china from 2014 to 2018.The label is heart failure. The age, gender and label distribution were shown in Fig. 10 (See the Additional file 1: Appendix for the specific attributes of the data set).

**Fig. 11:**
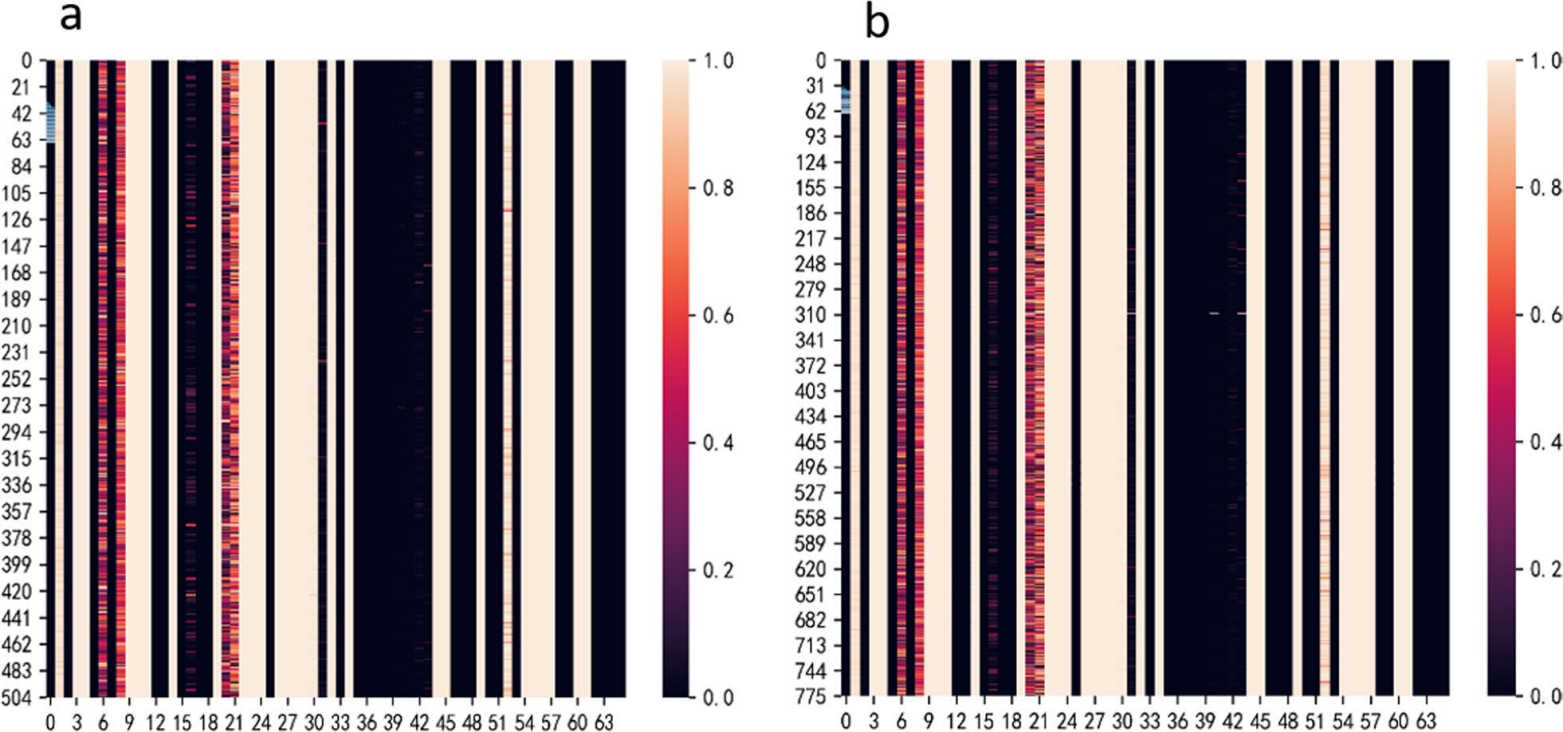
Female and male features selected for average probability heat maps. **a** Female, **b** male

As can be seen in Table 4, there is a slight performance improvement when discarding all of the irrelevant features. However, we can get which features the model prediction focuses on from the feature selection probabilistic heat map. Figure 11 depicts a heat map of the average probability of features selected for heart failure in male and female patients. It is concluded from the map that the male and female models focus on the same features.

**Table 4:** Prediction performance results

Datasets	Method	AUROC	AUPRC	ACC
Heart failure	XGBOOST	0.90 ± 0.04	0.792 ± 0.02	0.870 ± 0.06
	LR With LASSO	0.91 ± 0.03	0.723 ± 0.02	0.90 ± 0.11
	Neural network	0.912 ± 0.02	0.791 ± 0.02	0.899 ± 0.01
	SVM	0.881 ± 0.01	0.781 ± 0.02	0.851 ± 0.02
	With our model	0.924 ± 0.04	0.808 ± 0.02	0.90 ± 0.06

**Fig. 12:**
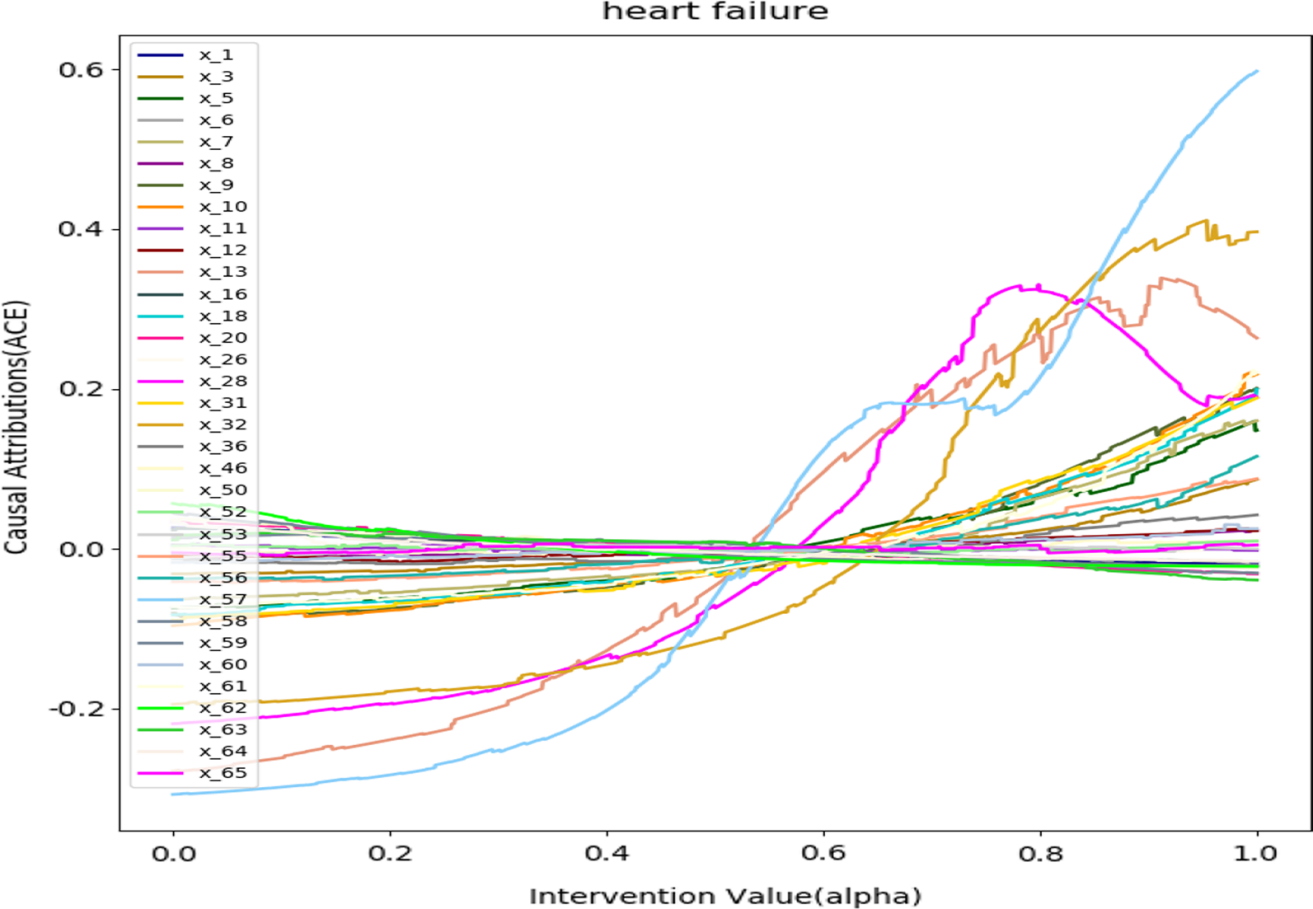
Average causal effect in patients with heart failure

Figure 12 depicts the causal effect of patient selection feature. As we can see from the figure that when the patient value is in the middle, the causal effect on the prediction of heart failure is not obvious. Because the value is in the normal range. When the patient's value is at both ends, the causal effect value changes significantly. In particular, the variables x_13, x_28, x_32, x_57 have a greater impact on the prediction of the patient. x_13 is the Direct bilirubin (DBIL). x_28 is the patient's intraoperative pulse variance. x_32 is the variance of the patient's intraoperative spo2. x_57 is the variance of the patient's intraoperative heart rate. The figure reveal that the larger x_28,x_32 and x_57 are positively causal (ACE ≥ 0) for heart failure. The analysis of the model is consistent with common medical knowledge. In addition, patient’s direct bilirubin is also positively causal for heart failure. We analyzed that the patient may have liver disease, which can lead to heart problems.

## Discussion

Traditional interpretability mainly focuses on statistical interpretability, while causal interpretability aims to answer questions related to causal intervention interpretability and counterfactual interpretability. For instance, traditional machine interpretability frameworks are not capable to answer causal questions such as “What is the impact of the nth filter of the mth layer of a deep neural network on the predictions of the model?” which are helpful and required for understanding a neural network model. Chattopadhyay et al. [8] propose an attribution method based on the first principle of causality. The proposed framework models the structure of the machine learning algorithm as an SCM. It then proposes a scalable causal inference approach to the estimate individual treatment effect of a desired component on the decision made by the algorithm. Therefore, we propose a two-stage prediction method (instance feature selection prediction and causal effect analysis) for instance disease prediction base on this work. The results of our experiments on synthetic data, open source data and real medical data show that our proposed method can provide qualitative and quantitative causal explanations for the model while giving prediction results.

The limitation of this work is that we only focus on the static attribute data of patients, while the model cannot deal with the clinical time series data. Future work will include extending to apply in the temporal setting. One such avenue of exploration for this would be to replace each of the networks with an RNN. This method can apply to medical time series data. Importantly, we believe this work can encourage viewing medical and health issues from a causal lens, and answering further causal questions such as: which counterfactual questions might be asked and answered in a medical and health issues, can a causal chain exist in medical and health issues and so on.

## Conclusions

This work presented a new causal perspective to feature selection and prediction. We propose a two-stage prediction method for instance disease prediction. Firstly, qualitative feature selection is performed on patients. The method is based on counterfactual and uses a reinforcement learning framework to design an interpretable instance feature selection prediction model. The methods of quantitative feature analysis views a neural network as an Structural Causal Model (SCM)to calculate the Average Causal Effect (ACE) of selected features in neural networks. The experiments on synthetic, open source, and real data show that the method can effectively select patient attributes for prediction and elicit causal effect of input on output data in neural networks.

## Supplementary Information

**Additional file 1**. Neural Network Attribution Related Definition. Definition of TPR and FDR. Data Set Attributes.

## Data Availability

The data of this experiment comes from three parts: synthetic data, open source data and real medical data. Synthetic data is automatically generated by computer based on formula. Obesity levels based on eating habits and physical condition Data Set came from the kaggle competition. It can be downloaded from the website (https://www.kaggle.com/ankurbajaj9/obesity-levels). The real medical data of patients with heart failure comes from the cooperative unit (Southwest Hospital).The raw data required to reproduce these findings cannot be shared at this time as the data also forms part of an ongoing study. If someone wants to reasonable request the data, you may contact the corresponding author.

## Abbreviations

ACE: Average causal effect
SCM: Structural causal model
AI: Artificial intelligence
SVM: Support vector machine
FCBF: Fast correlation-based filter
MRMR: Minimum redundancy and maximum relevance
