# Supplementary material for: Interpretable instance disease prediction based on causal feature selection and effect analysis
Source: BMC Med Inform Decis Mak. 2022 Feb 26;22:51. doi: 10.1186/s12911-022-01788-8 (PMC8881866; doi:10.1186/s12911-022-01788-8)
Supplement: Supplementary file 1 — Additional file 1. Neural Network Attribution Related Definition. Definition of TPR and FDR. Data Set Attributes. [file 12911_2022_1788_MOESM1_ESM.docx]

**Appendix**

**Neural network attribution related definition**

**Proposition 1**

An l-layer feed-forward neural network N$(l_{1},l_{2,}\ldots..l_{n})$ where $l_{i}$ is the set of neurons in layer i has a corresponding SCM M$([\left( l_{1},l_{2,}\ldots..l_{n} \right],U,\left[ f_{1},f_{2},\ldots.f_{n} \right],P_{U})$, where$l_{1}$ is the input layer and $l_{n}$ is the output layer. Corresponding to every$l_{i}$, $f_{i}$ refers to the set of causal functions for neurons in layer i. U refers to a set of exogenous random variables which act as causal factors for the input neurons $l_{1}$.

**Corollary 1**

Every l-layer feed-forward neural network N$(l_{1},l_{2,}\ldots..l_{n})$, with $l_{i}$ denoting the set of neurons in layer i, has a corresponding SCM M$([\left( l_{1},l_{2,}\ldots..l_{n} \right],U,\left[ f_{1},f_{2},\ldots.f_{n} \right],P_{U})$ which can be reduced to an SCM $M^{'}$ SCM M$([\left( l_{1},l_{n} \right],U,\left[ f_{0} \right],P_{U})$.

**Definition 1 (Average Causal Effect).**

The Average Causal Effect (ACE) of a binary random variable x on another random variable y is commonly defined as$E\left[ y | \mathrm{do}\left( x=1 \right) \right]-E[y|do(x=0)]$

**Definition 2 (Causal Attribution).**

We define ${ACE}_{do(x_{i}=\alpha)}^{y}$ as the causal attribution of input neuron $x_{i}$ for an output neuron y.

**Proposition 2**

Given an l-layer feed-forward neural network N$(l_{1},l_{2,}\ldots..l_{n})$ with $l_{i}$ denoting the set of neurons in layer i and its corresponding reduced SCM $M^{'}$ SCM M$([\left( l_{1},l_{n} \right],U,\left[ f_{0} \right],P_{U})$, the intervened input neuron is d-separated from all other input neurons.

**Corollary 2**

Given an l-layer feed-forward neural network N$(l_{1},l_{2,}\ldots..l_{n})$ with li denoting the set of neurons in layer i and an intervention on neuron $x_{i}$ , the probability distribution of all other input neurons does not change, i.e. $\forall x_{j}\in l_{1}$ and$x_{j}=x_{i} P\left( x_{j} | do\left( x_{i}=\alpha\right) \right)=P(x_{j})$.

**Definition of TPR and FDR**

|  | | True | |
| --- | --- | --- | --- |
|  |  | Positive | Negative |
| predicted | Positive | True  Positive | False  Positive |
|  | Negative | False  Negative | True  Negative |

True Positive Rate$=\frac{True Positive}{True Positive+False Negative}$

False Negative Rate=$\frac{False Positive}{True Positive+False Positive}$

Table 1. Obesity levels Data Set attributes

| **Num** | **attributes** |
| --- | --- |
| **1** | Frequent consumption of high caloric food(FAVC) |
| **2** | Frequency of consumption of vegetables (FCVC) |
| **3** | Number of main meals (NCP) |
| **4** | Consumption of food between meals (CAEC) |
| **5** | Consumption of water daily (CH20) |
| **6** | Consumption of alcohol (CALC). |
| **7** | Calories consumption monitoring (SCC) |
| **8** | Physical activity frequency (FAF) |
| **9** | Time using technology devices (TUE) |
| **10** | Time using technology devices (TUE) |
| **11** | Gender |
| **12** | Age |
| **13** | Height |
| **14** | Weight |

Table 2.Heart Failure Data Set attributes

| **Num** | **attributes** | **Num** | **attributes** |
| --- | --- | --- | --- |
| **1** | age | **2** | gender |
| **3** | Alanine aminotransferase (ALT) | **4** | Neutrophils % (Neut%) |
| **5** | Neutrophils count (Neut#) | **6** | International Standardized Ratio of Prothrombin (INR) |
| **7** | Prothrombin time (PT) | **8** | Aspartic acid aminotransferase (AST) |
| **9** | Urea | **10** | Activated partial thromboplastin time (APTT) |
| **11** | White blood cell count (WBC#) | **12** | Albumin (ALB) |
| **13** | Albumin/globulin ratio (Alb/Glo) | **14** | Direct bilirubin (DBIL) |
| **15** | RBC specific volume (HCT) | **16** | RBC Count (RBC#) |
| **17** | Fibrinogen (FBG) | **18** | Creatinine (CREA) |
| **19** | Bilirubin (Bil) | **20** | Glucose (Glu) |
| **21** | Hemoglobin (Hb) | **22** | Calcium (Ca) |
| **23** | Sodium (Na) | **24** | Potassium (K) |
| **25** | Indirect bilirubin (IBIL) | **26** | PULSE |
| **27** | PULSE_mean | **28** | PULSE_range |
| **29** | PULSE_std | **30** | SpO2 |
| **31** | SpO2_mean | **32** | SpO2_range |
| **33** | SpO2_std | **34** | last_time |
| **35** | Central venous pressure(CVP) | **36** | Central venous pressure _mean(CVP_mean) |
| **37** | Central venous pressure _range(CVP_range) | **38** | Central venous pressure _std(CVP_std) |
| **39** | Arterial mean pressure(AMP) | **40** | Mean arterial pressure _mean(AMP_mean) |
| **41** | Arterial systolic pressure _ range(AMP_range) | **42** | Arterial systolic pressure _ std(AMP_std) |
| **43** | Arterial systolic pressure(ASP) | **44** | Arterial systolic pressure _mean(ASP_mean) |
| **45** | Arterial systolic pressure _range(ASP_range) | **46** | Arterial systolic pressure _std(ASP_std) |
| **47** | Arterial diastolic pressure(ADP) | **48** | Arterial diastolic pressure _mean(ADP_mean) |
| **49** | Arterial diastolic pressure _range(ADP_range) | **50** | Arterial diastolic pressure _std(ADP_std) |
| **51** | Breathing(Bre) | **52** | Breathing _mean |
| **53** | Breathing _range | **54** | Breathing _std |
| **55** | Heart rate(HR) | **56** | Heart rate _mean(HR_mean) |
| **57** | Heart rate _range(HR_range) | **58** | Heart rate _std(HR_std) |
| **59** | Systolic blood pressure(SBP) | **60** | Systolic blood pressure _mean(SBP_mean) |
| **61** | Systolic blood pressure _range(SBP_range) | **62** | Systolic blood pressure _std(SBP_std) |
| **63** | Diastolic blood pressure(DBP) | **64** | Diastolic blood pressure _mean(DBP_mean) |
| **65** | Diastolic blood pressure _range(DBP_range) | **66** | Diastolic blood pressure _std(DBP_std) |
